# The World's Most Isolated and Distinct Whale Population? Humpback Whales of the Arabian Sea

**DOI:** 10.1371/journal.pone.0114162

**Published:** 2014-12-03

**Authors:** Cristina Pomilla, Ana R. Amaral, Tim Collins, Gianna Minton, Ken Findlay, Matthew S. Leslie, Louisa Ponnampalam, Robert Baldwin, Howard Rosenbaum

**Affiliations:** 1 Sackler Institute for Comparative Genomics, American Museum of Natural History, New York, New York, United States of America; 2 Centro de Biologia Ambiental, Faculdade de Ciências Universidade de Lisboa, Lisboa, Portugal; 3 Ocean Giants Program, Wildlife Conservation Society, Bronx, New York, United States of America; 4 Environment Society of Oman, Ruwi, Sultanate of Oman; 5 World Wide Fund for Nature, Gabon, Libreville, Gabon; 6 Mammal Research Institute, Department of Zoology and Entomology, University of Pretoria, Pretoria, South Africa; 7 Institute of Ocean and Earth Sciences, University of Malaya, Kuala Lumpur, Malaysia; University of Calgary, Canada

## Abstract

A clear understanding of population structure is essential for assessing conservation status and implementing management strategies. A small, non-migratory population of humpback whales in the Arabian Sea is classified as “Endangered” on the IUCN Red List of Threatened Species, an assessment constrained by a lack of data, including limited understanding of its relationship to other populations. We analysed 11 microsatellite markers and mitochondrial DNA sequences extracted from 67 Arabian Sea humpback whale tissue samples and compared them to equivalent datasets from the Southern Hemisphere and North Pacific. Results show that the Arabian Sea population is highly distinct; estimates of gene flow and divergence times suggest a Southern Indian Ocean origin but indicate that it has been isolated for approximately 70,000 years, remarkable for a species that is typically highly migratory. Genetic diversity values are significantly lower than those obtained for Southern Hemisphere populations and signatures of ancient and recent genetic bottlenecks were identified. Our findings suggest this is the world's most isolated humpback whale population, which, when combined with low population abundance estimates and anthropogenic threats, raises concern for its survival. We recommend an amendment of the status of the population to “Critically Endangered” on the IUCN Red List.

## Introduction

Understanding the patterns underlying the division of natural populations into smaller units is essential for the conservation and management of biodiversity. This is particularly relevant for species and populations that have been extensively exploited and require specific recovery measures. Many baleen whale populations were dramatically reduced by whaling [1], [2], with important consequences for their distribution, connectivity and genetic diversity. Some populations show signs of recovery (e.g. [3], [4] whilst others, e.g. Northern Hemisphere right whales, remain very small [5], [6], [7], [8], [9]). Their lack of recovery is due in part to modern anthropogenic threats, including entanglement in fishing gears and ship strikes (e.g. [10], [11]).

Humpback whales (*Megaptera novaeangliae*) were among those species heavily impacted by whaling, particularly in the Southern Hemisphere [12]. Distributed worldwide, they typically undertake long migrations between high latitude feeding grounds and low latitude breeding grounds, to which they show a high degree of site fidelity (e.g. [13]). Frequently coastal in their distribution, they were a preferred whaling target with all populations heavily impacted [6], [12]. Following the International Whaling Commission's (IWC) 1966 ban on commercial hunting of this species, many populations have begun to recover. Recent work has identified significant population structure among breeding concentrations in the North Pacific, North Atlantic and Southern Oceans [14], [15], [16]. Patterns of differentiation have been extensively studied in Northern (e.g.[14], [17]) and some Southern Hemisphere populations (e.g.[16]), but understanding of population structure and gene flow in the Indian Ocean remains limited, particularly for humpback whales in the Arabian Sea (Figure S1).

Historical records of Arabian Sea humpback whales (ASHW) indicate a distribution extending from Iraq [18], [19], to Iran, Pakistan, India, Oman and Yemen [20]. Gervais [19] believed that a damaged skeletal specimen from the Arabian Gulf was from a new species of *Megaptera* (*M. indica*) based on morphology. Other observers believed that ASHW were Southern hemisphere migrants [21], [22] whilst Slijper *et al.* [23] suggested they might be from the North Pacific [24]. Recent research efforts in the area confirm the continued presence of humpback whales in waters of Oman, Iran and Pakistan with highest reported encounter rates in the Gulf of Masirah and Kuria Muria Bay (both in Oman) [25], [26], [27], reflecting recent research efforts in the region. With the exception of whaling records [28], published observations in waters other than Oman are limited [29], [30].

Several lines of evidence indicate that ASHW form a discrete, small population: 1) their breeding cycle is more typical of Northern Hemisphere populations with observations of singing between January and March and peak calving in March [26], [28], [31]; 2) no whales photo-identified in Oman have been matched to research catalogues from Madagascar, South Africa, Mozambique or Zanzibar (suggesting little or no current migration) [26], [32]; 3) ASHW carry fewer and smaller barnacles and barnacle scars than whales in other localities and 4) do not exhibit cookie cutter shark (*Isistius brasiliensis*) bites, commonly seen on some Southern Hemisphere whales [31]; 5) whale songs recorded in Oman between 2000–2003 are highly distinct from those recorded concurrently in Madagascar ([26], [33]; S. Cerchio, Pers. Comm.] and 6) photographic mark-recapture (MR) data for whales sighted in Oman indicated seasonal movements for some individuals between Dhofar (Hallaniyat Islands, Kuria Muria Bay) in late winter/spring and the Gulf of Masirah (and probably Sea of Oman) in autumn [26] indicating a likely year-round residence off the coast of Oman. The same MR data, collected between 2000 and 2004, yielded an abundance estimate of 82 individuals (95% CI = 60–111), although numerous caveats were noted [26].

The species is globally classified as “Least Concern” on the IUCN Red List of Threatened Species, but the ASHW population is classified as “Endangered”, an assessment based on contemporary scientific evidence and information on regional threats [20]. The small MR abundance estimate was insufficient to justify an assessment of “Critically Endangered” as sample sizes were small and much of the putative range was not surveyed (introducing considerable potential downward biases to the abundance estimate across the region).

**Figure 1:**
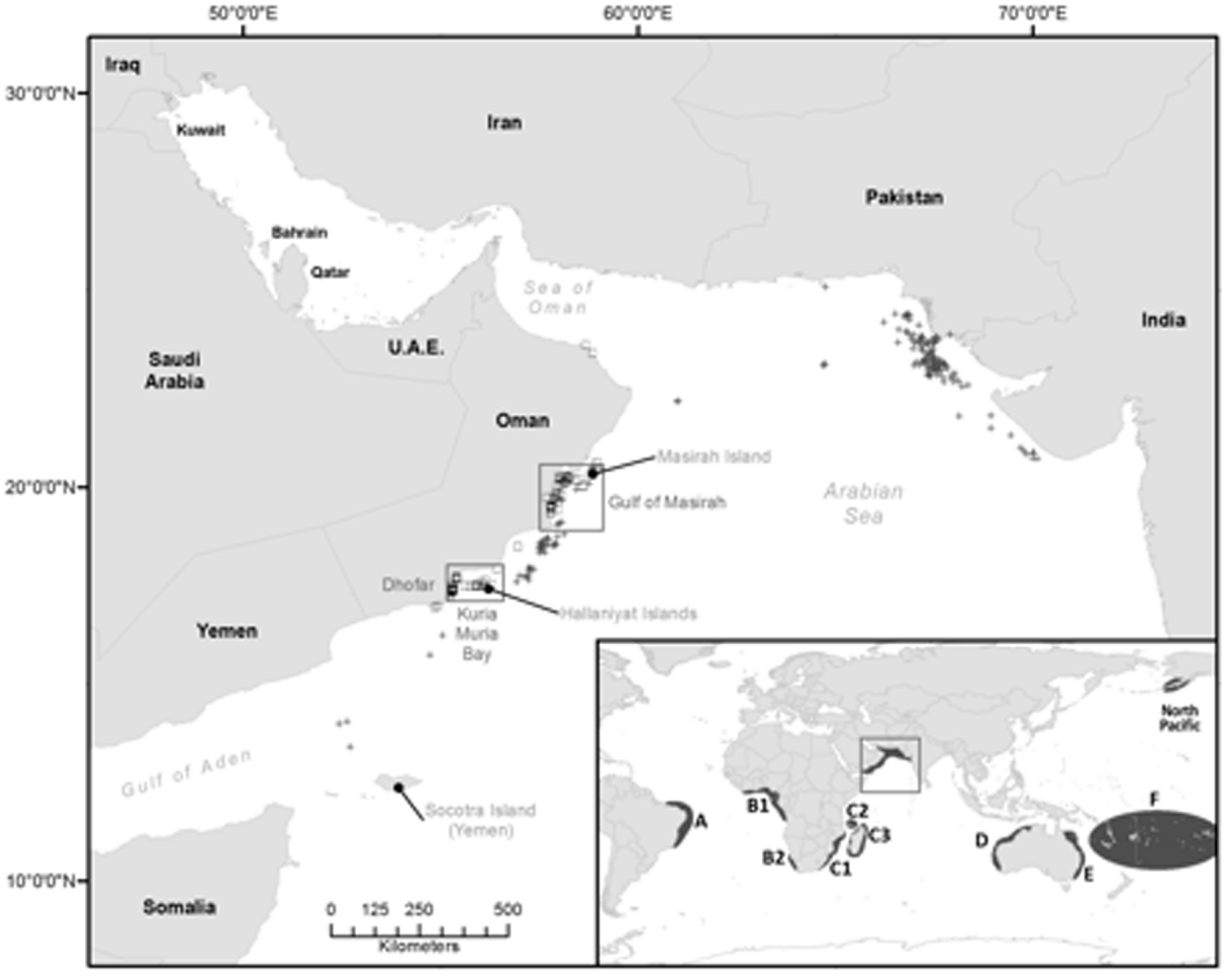
Main panel: the Arabian Sea region showing sampling locations in Oman (boxed regions) as well as locations of Soviet catches from the 1960's (crosses) and modern sightings from Oman (squares). Inset: sampling locations used in this study, including six Southern Hemisphere breeding Stocks (A–F), the North Pacific and the Arabian Sea (boxed region).

The extent to which ASHW are related to whales in other populations is not well understood. The IWC has defined seven Southern Hemisphere humpback whale breeding ‘Stocks’ (A–G, see Figure 1 inset), and we use these designations hereafter to describe them. Recent mitochondrial DNA (mtDNA) analyses found strong genetic structure between the ASHW and IWC breeding Stocks A and B in the South Atlantic Ocean and IWC Breeding Stock C in the south-western Indian Ocean (pair-wise FST range between Oman and other Indian Ocean breeding populations 0.11–0.15, 33) [16]. This study however did not test connections between ASHW and the eastern Indian Ocean, or investigate Northern Hemisphere links.

In this paper we combine mtDNA and nuclear microsatellite data with traditional and coalescent-based statistical analyses of population structure to: i) comprehensively test hypotheses on the origin and inter-population connections, if any, of ASHW; and ii) assess population status from a conservation genetics point of view. To address these objectives we used data from four Indo-Pacific Breeding Stocks (IWC Breeding Stocks C, D, E, F, see Figure 1 inset), the North Pacific, and two South Atlantic Breeding Stocks (A and B), regions with the most ‘feasible’ migration links to the Arabian Sea. We found that the ASHW population is genetically highly differentiated from Southern Hemisphere populations. This evidence, coupled with a lack of current migrants and a small population size, make this population the world's most isolated and distinct humpback whale population. We believe its conservation status should be raised to “Critically Endangered” on the IUCN Red List of Threatened Species.

This study not only contributes to our understanding of the demographic and evolutionary patterns that have shaped population structure and gene flow in this highly migratory species but also has important implications for its conservation and management.

## Materials and Methods

### Ethics statement

All research undertaken followed local regulations and guidelines. The project was also approved by the American Museum of Natural History Institutional Animal Care and Use Committee (IACUC). Biopsies were taken under permits numbers 10/2000, 6/2002 and 07/2004, issued by the Directorate - General of Nature Conservation of the Ministry of Regional Municipalities, Environment and Water Resources of the Sultanate of Oman. CITES permits were issued by the same institution (permit numbers 3/2002, 13/2002, 1/2003 and 7/2003).

### Sample collection and DNA extraction

Tissue samples were collected during small-boat surveys between 1999 and 2004. Surveys were conducted in the Gulf of Masirah and the Dhofar coast of Oman (Figure 1). Surveys occurred between October and March and targeted areas where published [28] and unpublished records indicated the presence of whales. Tissue samples (n = 67) were mostly obtained using the biopsy dart procedure [34], as sloughed skin (n = 14, including two from net-entangled whales that were released) and from stranded animals (n = 5). Samples were preserved in salt saturated 20% Dimethyl Sulfoxide solution (DMSO) and stored at −20°C until processed. Total genomic DNA was extracted from the epidermal layer using the DNAeasy tissue kit (Qiagen).

### Mitochondrial DNA and microsatellite molecular analyses

A 520 bp fragment within the mtDNA control region was amplified with primers Dlp-1.5 and Dlp-5 [35]. Polymerase Chain Reaction (PCR) products were cycle-sequenced (forward and reverse) with dye-labelled terminators using manufacturers recommendations. Sequence reactions were analysed using an ABI-Prism 3700 or 3730 DNA Analyser (Applied Biosystems). Sequence variation and polymorphism analyses are detailed in the Supporting Information.

Eleven cetacean di-, tri- and tetra- nucleotide microsatellite loci were selected: 199/200, 417/418 and 464/465 [36], EV1Pm, EV37Mn, EV94Mn and EV96Mn [37], and GATA028, TAA031, GATA053 and GATA417 [38]. One primer of each pair was labelled with a fluorescent tag (HEX, 6-FAM and TET, Qiagen-Operon; NED, Applied Biosystems) on the 5′ end. Polymerase chain reactions (PCRs) were carried out in a 20µl or 10µl volume with the following conditions: 50 mM KCl, 10 mM Tris-HCl pH8.8, 2.5–3.5 mM MgCl_2_, 200µM of each dNTP, 0.4µM of each primer, 0.025 U/µl *Taq* Gold polymerase (Perkin-Elmer). Amplifications were completed in either a Perkin-Elmer 9600 thermal cycler or an Eppendorf Gradient Mastercycler, after optimization of published annealing temperatures and profiles. Pooled PCR products were loaded with the addition of an internal standard ladder (Genscan-500 TAMRA or ROX, Applied Biosystems) on a 3700 or 3730 DNA analyzer (Applied Biosystems). The allele size in base pairs was identified with the software GeneScan Analyses and Genotyper 2.1 or Genemapper (Applied Biosystems). Hardy-Weinberg equilibrium (HWE) and linkage disequilibrium (LD) at microsatellite loci were evaluated with a probability test [39] implemented in Genepop v.3.4 [40].

### Statistical analysis

Duplicate samples were detected using genotype identity with the Excel add-in MS_Toolkit package [41]; they were then eliminated. The average probability of different random individuals sharing the same genotype by chance (Probability of Identity, PI) was estimated using Api-Calc 1.0 [42].

From the 520 bp mtDNA fragment, a 486 bp consensus region that contains the majority of variable nucleotide positions in the mtDNA control region of humpback whales was examined for all samples [43]. Sequences were aligned and edited using Sequencher v. 4.5 (Gene Codes Corp. Ann Arbor, MI). Comparisons of sequences to identify polymorphic sites and haplotypes were performed using Macclade v. 4.01 [44].

### Origin of the ASHW population

To infer possible relationships of ASHW with other populations we analysed mtDNA control region sequences for 740 individuals sampled from across IWC Breeding Stock C. We obtained sequences for 174 samples (464 bp) from GenBank for IWC Breeding Stock D [45], 605 samples (464 bp) for IWC Breeding Stock E, 230 samples (464 bp) for IWC Breeding Stock F [45] and 54 samples (425 bp) from the North Pacific [46]. Eleven microsatellite markers obtained for 1531 individuals sampled from IWC Breeding Stocks A and B (Brazil, Gabon, Angola and West South Africa) and IWC Breeding Stocks C1, C2 and C3 (East South Africa, Mozambique, Mayotte, Madagascar) were also analysed, in order to understand the level of genetic differentiation in populations geographically closer to ASHW (Figure 1 inset).

A phylogeny of the humpback whale mtDNA haplotypes identified from all sequences listed above was constructed using the Bayesian Inference method as implemented in MrBayes v. 3.2. [47]. The sequences were adjusted for multiple substitutions using the Kimura 2-parameter model [48]. A fin whale sequence from GenBank was used as an outgroup. Four simultaneous Markov chain Monte Carlo (MCMC) chains were run for 7 × 10^6^ generations, with trees sampled at intervals of 1,000 generations. The first 5,000 trees were discarded as burn-in after examining the variation in log-likelihood scores over time.

The differentiation between the Arabian Sea and the other areas was quantified using pairwise *F*-statistics, implemented in Arlequin v 3.11 [49]. These were calculated for mtDNA nucleotide differentiation (*Φ_ST_*) and haplotype frequency differences (*F_ST_*), as well as for microsatellite allele frequency differences (*F_ST_*). The significance of the observed *Φ_ST_* and *F_ST_* values was tested using 10,000 random permutations of the data matrix. No correction for simultaneous tests was applied to significance levels of pairwise comparisons [50].

Population subdivisions were tested using microsatellite data and a Bayesian model-based clustering method implemented in Structure v.2.3.4. [51]. This method assumes the presence of *K* populations, where *K* may be unknown and uses a MCMC procedure for its estimation. We used the ‘correlated frequencies' model, excluding admixture and using prior information on sample origin (‘locprior option) (these models are more powerful at detecting subtle population structure [52]) and also without supplying prior information on sample origin (2×10^5^ “burn in” and 2×10^6^ chain iterations). The true number of populations was estimated in Structure Harvester [53] as the highest averaged posterior probability (ln Pr(*X*|*K*)) of the data. *CLUMPP* V.1.1.2 [54] was used to summarize parameters across the 10 runs and *distruct* v.1.1 [55] was used to produce the corresponding graphical output.

In order to estimate effective migration rates and divergence times between ASHW and other areas we analysed mtDNA data using the program Mdiv [56], which uses a MCMC method within a likelihood framework to estimate the posterior distributions of the migration rate per gene per generation [M = (2 *N_e_*m)], the time in generations since the two populations diverged scaled by the effective population size [T = (t/2 *N_e_*)], and the parameter theta (θ), which is a product of the effective population size and the mutation rate per generation of the studied gene region [θ = (4 *N_e_*μ)]. Default maximum values for Mmax and Tmax were used. A minimum of five runs was carried out for each comparison and results were averaged between runs (10^6^ dememorization iterations, 5×10^6^ chain iterations). Terminal ends of sequences were truncated as they contained gaps, which Mdiv is unable to handle.

### Status of the ASHW population

The diversity of humpback whale mtDNA sequences was estimated as haplotype diversity (*Hd*), and mean number of pairwise nucleotide differences (*k*) [57], [58] using Arlequin v.3.11 [59]. For nuclear microsatellites the mean number of alleles per locus (*K*), the observed heterozygosity (*H*_o_), and heterozygosity expected (*H*_e_) under Hardy-Weinberg assumptions [57] were calculated in Fstat v.2.9.3 [60].

To test for population size reduction we used two approaches. We calculated the value *M*, the ratio between the number of alleles and the range of the allelic array, using the software M-Ratio [61]. *M* declines after a population reduces in size and the magnitude of the decrease is positively correlated with the severity and duration of the reduction. A simulation approach was used to calculate a critical value for the ratio *M* (*M*c) in an equilibrium population, below which one can assume that a data set is a sample from a population that has experienced a recent size reduction. We ran the simulation under a conservative two-phase mutation model where the proportion of one-step mutations is *p_s_* = 90% and the average size of nonone-step mutations is Δ*_g_* = 3.5, as well as a realistic model obtained from published data (*p_s_* = 88%, Δ*_g_* = 2.8) [61]. The simulations were repeated for different values (from 0.004 to 5) of historical diversity θ (4 *N_e_*μ), representing different equilibrium population sizes if we assume a constant *µ* (e.g. 5 × 10-4, [61]). The recovery rate of *M* is positively correlated with post-reduction population size, but that recovery occurs in both small and large populations, indicating that *M* can distinguish between populations that have been recently reduced in size and those that have been small for a long time. In addition we used Bottleneck v. 1.2.02 [62] to test for allele frequency mode shifts (variation from the typical L-shape distribution). For this test, observed genetic diversity was compared with expected equilibrium gene diversity (10,000 simulations) by means of a one-tailed Wilcoxon test.

To further investigate population stability we applied three tests of the neutral theory of molecular evolution to the mtDNA data. The raggedness statistic *rg* analyses the distribution of pairwise differences, or mismatch distribution [63], [64]; Tajima's *D* [65] and *R*_2_ statistics [66] are both based on the distribution of mutation frequencies. Test significance was assessed with coalescent simulations. All tests and simulations were conducted with Dnasp v.4.10.9 [59]. In addition we constructed Bayesian Skyline Plots (BSP), which use the coalescent theory to infer changes in effective population size through time [67]. The BSP was constructed using Beast v. 1.7.4 [68], with MCMC runs of up to 10 million steps that yielded effective sample sizes (ESS) of at least 200. A mutation rate of 5.2% per million years was used [69].

In order to estimate contemporary effective population size (*Ne*) for the ASHW using the microsatellite data, we used two methods as implemented in the program NeEstimator V2 [70]: a method based on linkage disequilibrium (LD) [71] and the heterozygote-excess method [72]. While the first method is based on random linkage disequilibrium that arises by chance each generation in finite population, the second method is based on the observation that when the number of breeders is very small, an excess of heterozygotes is expected in the progeny. Both these methods have proved to be quite powerful in estimating effective population sizes of small populations, as is the case of the ASHW. For comparison purposes, we have also estimated *Ne* for the other humpback whale populations analysed in the study, acknowledging that for larger populations, these estimates are not as accurate [71].

## Results

### Sample size and mtDNA and microsatellite variation

A total of 67 ASHW samples collected from Oman were included in the study. Four samples did not yield amplifiable DNA and were excluded, and for the remaining 63 samples the average Probability of Identity was small enough to exclude 16 re-samples with confidence; 47 individuals were included in the subsequent analyses. A consensus region of 486 bp of the mtDNA control region was assembled in which a total of 7 maternal haplotypes was detected from 24 polymorphic sites (GenBank Accession Numbers GQ913845, GQ913846, GQ913848, GQ913701, GQ913706, GQ913709, GQ913715). The 1,798 samples analysed from other populations yielded 167 haplotypes (GenBank Accession Numbers GQ913691-GQ913852). For microsatellites, missing allelic data averaged 0.3% across all loci, the largest number of alleles (12) was found at EV37Mn, the smallest (3) was recorded at locus EV1Pm. Deviation from Hardy-Weinberg equilibrium was rejected for all single loci and across loci (*p* = 0.79) and no significant heterozygote deficiency was found. In the same way there was no evidence of Linkage Disequilibrium.

### Origin of the ASHW population

**Figure 2:**
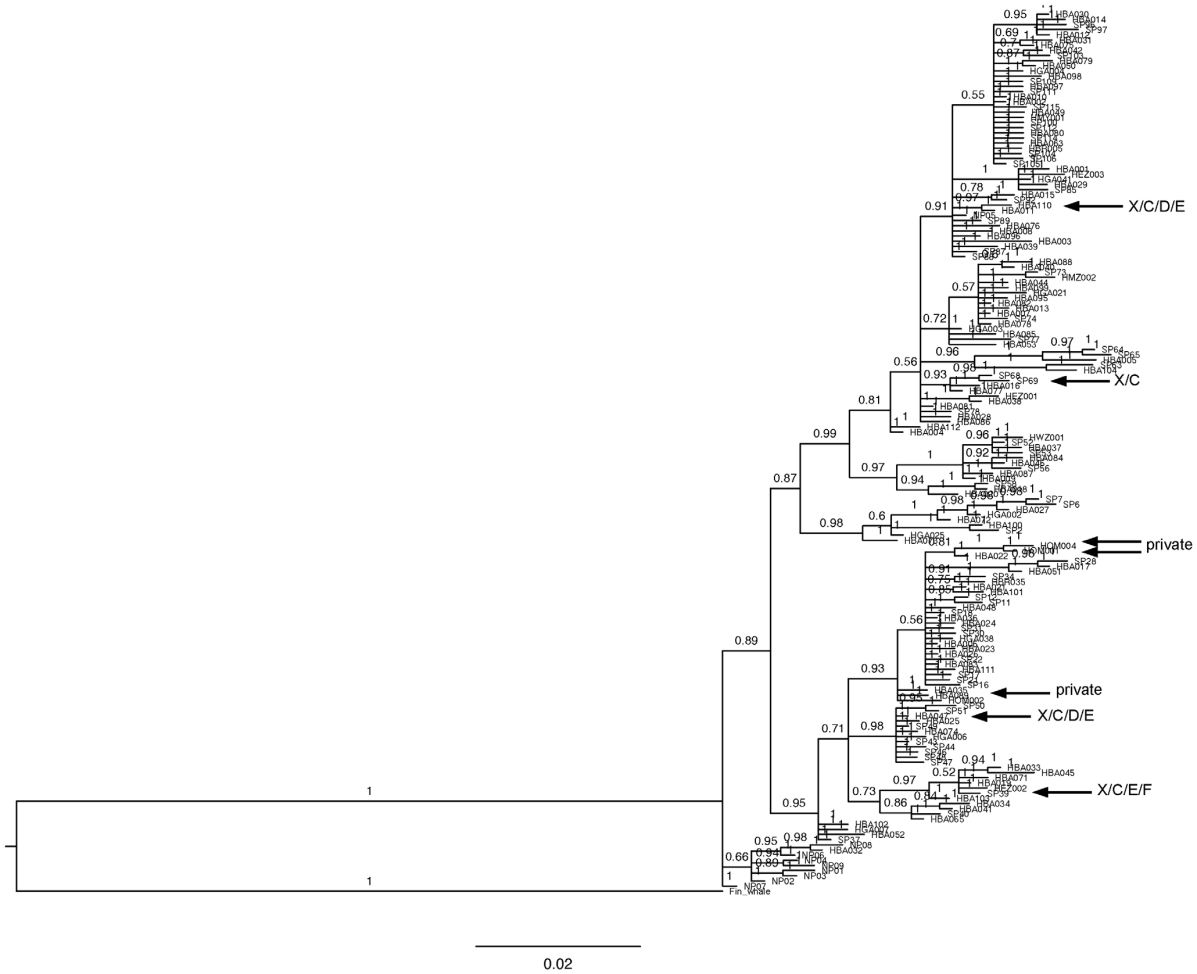
Bayesian phylogenetic tree constructed from 170 haplotypes from Stocks X, C, D, E, F and North Pacific. Stock X haplotypes are indicated by arrows.

Of the seven mtDNA haplotypes identified in the ASHW samples, one was shared with Stock C, one was shared with Stocks C and D, one was shared with Stocks C, D and E, one was shared with Stocks C, E and F, and three were private. In the phylogenetic tree (Figure 2) constructed from all 170 haplotypes plus the outgroup, the private ASHW haplotypes were placed in separate clades together with Southern Hemisphere haplotypes, the closest of which were from Stocks C and D. The North Pacific haplotypes formed a separate clade with the exception of one haplotype, which was shared with Southern Hemisphere samples (SP88).

**Figure 3:**
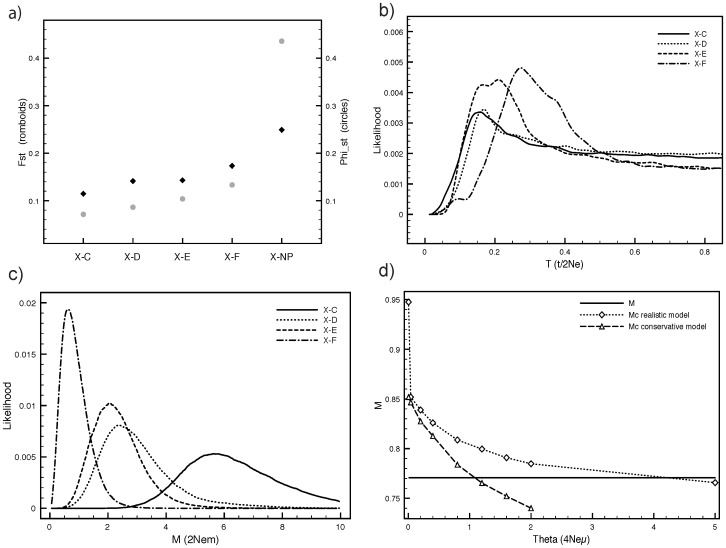
a) top left: pairwise measures of mtDNA differentiation between Stock X (Arabian Sea) and Stocks C to F in the Southern Hemisphere, and two sampling location in the North Pacific (grouped as NP). Rhomboids refer to *F*_ST_, while circles to *Φ*_ST_; b) top right: MDIV estimates of divergence time; c) bottom left: MDIV estimates of migration rates; d) bottom right: Bottleneck analyses [61]. M is the ratio between number of alleles and range of the allelic array. Mc is the minimum critical value for the ratio in an equilibrium population and for different historical diversity values (theta) calculated through simulations. The conservative two-phase mutation model assumes the proportion of one-step mutations to be ps = 90% and the average size of non one-step mutations Δg = 3.5. The realistic model is based on literature data (ps = 88%, Δg = 2.8).

Pairwise *F*-statistics showed high levels of differentiation for ASHW for both nuclear and mitochondrial markers and is the highest recorded for population differentiation of any humpback whale population worldwide [16]. For mtDNA (Figure 3a) the highest divergence was with the North Pacific, while comparisons with Southern Hemisphere Stocks showed a differentiation gradient related to geographic distance (C lowest, F highest). For microsatellites, comparisons between ASHW and five locations within Stock C showed strong structure, with the highest divergence from Sub-Stocks C2/C3 (*F*_ST_ =  0.046–0.048) and the lowest from Sub-Stock C1 (*F*_ST_ =  0.040). No genotypic matches were identified.

**Figure 4:**
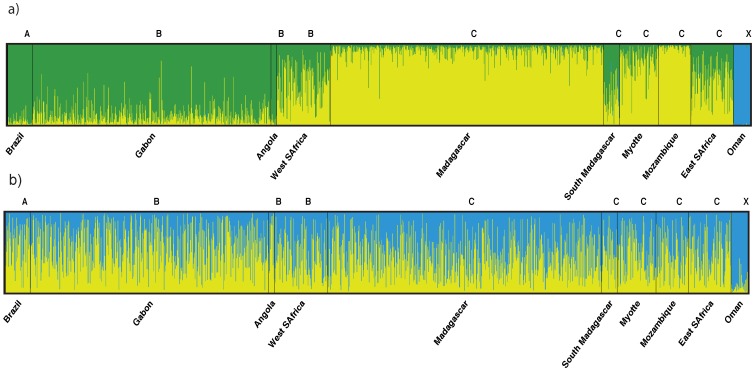
Results from STRUCTURE showing ancestry estimates for: a) *K*  =  3 (with ‘locprior’ option; and b) *K*  =  2 (without ‘locprior’ option). Each individual in the data set is represented by a single vertical line, which is partitioned into *K* colored segments that represent the estimated membership fraction of that individual in each of the *K* inferred clusters.

For Structure computations, the highest posterior probability of the data was obtained when the individuals were partitioned in three clusters when the ‘locprior’ option was used and in two clusters when it was not (Table S1). In both scenarios, all ASHW individuals were assigned to a distinct cluster (Figure 4a–b). The use of sampling origin prior is recommended to achieve better results when the amount of data is limited or the population structure is weak [52]. If we consider *K* = 3 as the most likely number of clusters in our dataset [52] all ASHW individuals have probabilities above 0.90 of belonging to the same cluster (Figure 4a). The two other clusters suggested by this analysis divide the remaining populations into two clear distinct groups comprising respectively most individuals from the Atlantic (Stocks A and B) and Indian Oceans (Stock C). Cluster identity is less clear where the two groups come in close contact (West and Eastern South Africa) and in Southern Madagascar, suggesting some degree of admixture for these populations (Figure 4a). If we consider *K* = 2 as the most likely number of clusters in our dataset, within the Arabian Sea population only a few individuals have probabilities around 0.5 of being assigned to the other cluster (Figure 4b). For all other sampling locations individuals have equal probabilities of belonging to any of the two clusters.

Mdiv coalescent analyses showed that ASHW diverged first from the North Pacific (T>0.2), and then from Southern Hemisphere Stocks. The closest divergence time is between ASHW and Stock C (Figure 3b, T = 0.1684). However given similar T values it cannot be excluded that ASHW diverged from C and D at about the same time. Using T, an average estimate for *θ* = 17.291, a mutation rate of 5.2% per million years[70], [74], a generation time of 21.5 years [73], and the analyzed sequence length (477 bp) we obtained a divergence time of ∼70,000 years ago.

The posterior distributions of migration rates showed that since divergence, gene flow between ASHW and the Southern Hemisphere has been limited. The highest exchange has been with Stock C (M = 5.67, Figure 3c). It is useful to note that M values between distinct contiguous stocks in the Southern Hemisphere are 5–6 times larger [16].

### Status of the ASHW population

**Figure 5:**
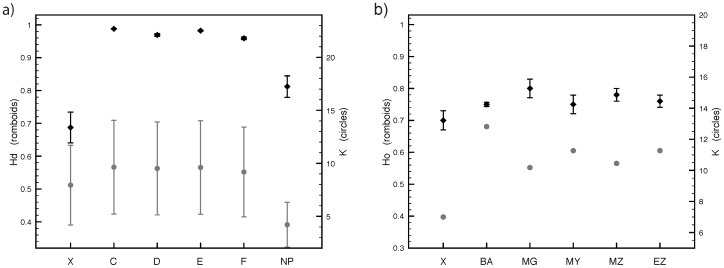
a) mtDNA diversity: rhomboids refer to haplotype diversity (*H*d), and circles to the mean number of pairwise differences (k); b) microsatellite diversity: rhomboids refer to the observed heterozygosity (*H*o), and circles to the mean number of alleles per locus (K). Bars represent the standard deviation.

Reduced genetic diversity for ASHW was confirmed by both mtDNA and microsatellite analyses (Figure 5). The mtDNA haplotype diversity (Hd) was lower than in any of the other areas analyzed. The average number of pairwise nucleotide differences (k) was larger only when compared to the North Pacific. Reduced diversity for Northern Hemisphere whales compared to Southern Hemisphere whales has been previously described [74]. For microsatellites both observed heterozygosity (Ho) and mean number of alleles per locus (K) were smaller for ASHW than for five Stock C sampling locations.

The observed ratio between the number of alleles and the range of the allelic array was *M* = 0.7706 (Figure 3d). Coalescent simulations of the data at equilibrium were carried out under a realistic mutation model for different historical diversity values (*θ* = 0.004 to *θ* = 5). The *M*-ratio produced by coalescent simulations (*M*c) was higher than that produced by the current data for *θ* <4.2, which corresponds to a pre-bottleneck effective population size of ∼2,100 animals (µ = 5 × 10^−4^) (Figure 3d). When the conservative mutation model was applied, this yielded an estimated cut-off value of *θ* ∼1.1, which corresponds to a pre-bottleneck effective population size of ∼550 animals. If the pre-bottleneck *N*_e_ was larger than these cut-off values there would be no evidence of a bottleneck (i.e. *M*c is smaller than *M*). Considering that effective population sizes correspond to ∼10% of census sizes [75], both these estimates are large enough to justify the assumption that the population experienced a bottleneck. *M* starts to decline immediately, within 1–2 generations after the bottleneck. Additionally, if a population stays small after a bottleneck event, it takes ∼300 generations for *M* to increase to normal levels [61]. This means that ASHW may have experienced a bottleneck as recently as 20 years ago, or as early as ∼6,450 years ago (using a generation time of 21.5 years).

In support of the above result, significant Tajima's *D* [*D* = 1.8574, *P(D*≥1.8574) = 0.023] and Ramos-Onsins and Rozas' *R*_2_ (*R*_2_ = 0.1763, *P(R*_2_≥0.1763) = 0.017) indicated that the population is not currently at equilibrium. These statistics generally test for population expansion, but when the observed value falls in the upper tail of the distribution, as it does for both tests, it may indicate population decline (J. Rozas, Pers. Comm.). Raggedness statistics (*rg* = 0.2566 *p* = 0.009) significantly rejected a population expansion.

Results from bottleneck indicate an excess of heterozygosity (one-tailed Wilcoxon test for heterozygosity excess, *P* <0.05), which suggests a recent reduction in effective population size of the ASHW. However, an analysis of the allele frequency distribution revealed an L-shaped distribution, which is expected for a population that has not experienced a recent bottleneck that affected genetic variability [76].

**Figure 6:**
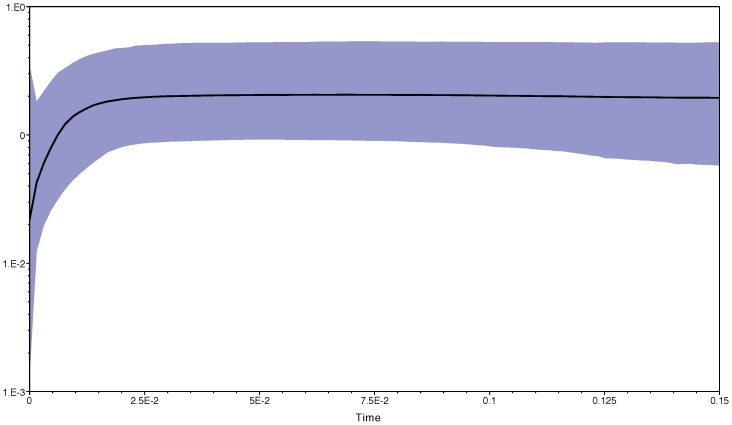
Bayesian skyline plot obtained with the mitochondrial DNA dataset, showing changes in effective population size through time (Ma).

The Bayesian skyline plot obtained in beast with the mitochondrial DNA shows a decline of effective population size through time, which according to the molecular clock used, may have started at around 15,000 y.a until the present day (Figure 6).

Estimates of contemporary effective population size (*N*e) for the ASHW resulted in values that range from 90.4 to 142.6 if we consider the method based on LD and different allele frequencies (Table S1). The number of breeders (*N*b) estimated with the heterozygote excess method ranged from 26.1 to 29.9 (Table S1). These figures are of the same order of magnitude as the estimate based on mark-recapture data. All other Southern Hemisphere humpback whale populations had much larger estimates of effective population size (Table S2), although these results should be interpreted with caution given the possible violation of assumptions due to the larger size of these populations, which may compromise the methods.

## Discussion

ASHW show strikingly high levels of genetic differentiation from Southern Hemisphere and North Pacific populations using mitochondrial and nuclear molecular markers. Our results suggest that this population likely originated as a consequence of a range expansion of whales from the Southern Indian Ocean dating ca. 70 kya. This timing also corresponds to the estimated radiation of a second clade from the Southern Hemisphere to the North Pacific [77] and we can speculate that these events were connected to the ongoing glaciation [78]. Similarities of the temperature histories of the last 65 kyr in the western Indian Ocean and Antarctica suggest a thermal coupling between the two regions on millennial timescales, conducive to an increased thermal stratification during southern hemisphere cold periods [79]. Although gene flow seems to have occurred after divergence, we consider it unlikely that migrants are currently being exchanged between the Arabian Sea and the Southern Indian Ocean stocks and shared mtDNA haplotypes may simply be the result of shared ancestry. An out-of-phase breeding cycle also acts as an effective barrier to interbreeding [24], [31], [80].

The switching of a non-migratory population to a Northern Hemisphere breeding cycle is an interesting biological conundrum and while this is beyond the scope of our research, it is tempting to hypothesize an adaptive response of the ASHW to the Arabian Sea seasonal cycle of temperature and productivity in connection to the monsoon seasons. Colder surface water, upwelling and primary productivity in the Arabian Sea is linked to the southwest (or ‘summer’) monsoon (May-September) while convective mixing and slightly warmer water is connected to the northeast (or ‘winter’) monsoon (October-March) [81]. Interestingly, evidence suggests that at the time of the estimated expansion from the Southern Indian Ocean, the Arabian Sea was entering a period of variable climatology (60–18 kya) in connection to the Last Glacial Age [82]. The shift of most productivity to the winter months during this period, due to significant weakened summer monsoons, but enhanced winter upwelling and nutrient supply from surrounding land caused by intense winter winds [82], would have suited the breeding cycle of whales from the Southern Hemisphere [85], [86] [24], [31], [82].

Our results further suggest that ASHW is constituted of remnant animals from a declining population. Reduced genetic diversity in both mtDNA and nuclear microsatellites, and evidence of recent bottlenecks support this hypothesis. Different bottleneck estimates were obtained with microsatellites (20-6,450 ya) and with mtDNA (15,000 ya), which may reflect marker characteristics; mtDNA is likely showing an older signal than the microsatellites. A primary productivity collapse occurred ∼15,000 ya, coinciding with the apparent signal of population decline in the mtDNA BSP. ASHW feed in monsoon-driven upwelling areas of Oman, Pakistan and India [28], [80], and a decline in food availability could be responsible for initiating a population decline.

Another minimum in primary productivity, consistent with the microsatellites bottleneck estimate (20-6,450 ya), is reported to have occurred 1,500–2,000 ya in correspondence to a weak summer monsoon [83]. Alternatively, the dating of the bottleneck could also be compatible with an episode of illegal Soviet whaling in the 1960's, despite the large confidence interval. Soviet records indicate that 242 ASHW were captured during brief episodes in both 1965 and 1966 (Figure 1). Soviet scientists estimated (subjectively) that this represented ∼60% of the whales they found [31]. A more recent bottleneck is further supported by tests of population expansion, which suggest the population may still be in decline, as is clearly seen in the BSP plot. This finding is of special concern if we consider several lines of evidence that suggest this population is spatially, genetically and demographically isolated from other breeding populations.

Scenarios of slow or no population recovery following whaling are seen in other whales species such as the Critically Endangered North Atlantic right whale (*Eubalaena glacialis* e.g. [7]) and Western gray whale (*Eschrichtius robustus*, [9]). However, populations of these species have larger estimated population sizes (∼500 North Atlantic right whales [84] and ∼130 Western gray whales [85]) and migrate between feeding and breeding grounds. The effective population size estimate for the ASHW obtained in this study suggests a population size of around 100 animals, which is in agreement with the census estimate of 82 individuals obtained for the area off Oman using mark-recapture data [20]. Estimates of effective population size using genetic data are prone to several errors due to sampling bias, variability of the genetic markers used and violation of assumptions such as that only drift is responsible for the signal in the data. Nevertheless, these effects are less likely to bias estimates of small populations like ASHW [71]. The small size of this population, the lack of migration and high level of genetic differentiation (the highest *F*_ST_ comparisons of any humpback whale population), make the ASHW the world's most isolated and most distinct population of humpback whales. Its uniqueness is comparable to another relict population, the eastern North Pacific right whale [8], a population that was drastically reduced by illegal Soviet whaling during the same period as potentially the ASHW and whose effective population size has remained low or declined over the last few decades [86].

Scarring analyses for ASHW identified in Oman in 2003 indicated that ∼30–40% of examined whales were likely to have been entangled in fishing gear [80]. This is a concern as fishing effort in Oman and the wider Arabian Sea is increasing rapidly (Ministry of National Economy, 2009; FAO, 2007). Furthermore, industrial ports, fast-ferry terminals and coastal resorts are under construction in Oman, with consequences for inshore ASHW habitats, including favoured breeding and feeding sites [80]. A further threat faced by ASHW is disease, which may be of particular concern given their low genetic diversity. A recent analysis showed a persistent occurrence of Tattoo Skin Disease (TSD) in 25.6% of 43 whales examined [87]. While not thought to be lethal, TSD infections may decrease overall fitness and increase vulnerability to other impacts [88], [89].

It is widely assumed that limited genetic diversity in small populations results in inbreeding depression; this can be manifest as the expression of deleterious alleles, or in a reduction of the genetic diversity that would normally provide immunity to epizootics or other threats [90], [91]. Amos [92] argues that human exploitation is unlikely to have significantly impacted genetic variability in cetaceans, but also that effects of inbreeding depression may be manifest in populations deemed from their levels of variability to be genetically ‘healthy’. Genetic diversity may well be relative; consequently, the critical level of diversity for a given population is often difficult to assess. Sufficient evidence confirms that if genetic factors are ignored, extinction risks can be underestimated and inappropriate conservation management strategies may be used [93]. However, there is also evidence to suggest that effective management and the reduction of threats to individual survival may reverse population decline, even if genetic variability is low [91].

All evidence collected and anlyzed suggests that ASHW are genetically and demographically unique, and exhibit atypical behaviour for humpback whales. The implementation of conservation and management measures for the ASHW population is of paramount importance and we recommend that the conservation status of ASHW be revised to “Critically Endangered” on the IUCN Red List of Threatened Species based on fulfilment of two criteria: 1) an estimated/suspected population size reduction of ≥ 80% over the last three generations, where the reduction or its causes may not have ceased or may not be understood; 2) population size estimated to number fewer than 250 mature individuals and an estimated continuing decline of at least 25% within one generation. According to the observations of the Soviet whalers the ASHW population size may have been at least 400 individuals 50 years (2–3 generations) ago. The current estimates for population size (82 individuals using mark-recapture data and 90–142 individuals using genetic data) and bottleneck timing are compatible with an 80% reduction. Additionally, our evidences of ongoing population decline indicate that while whaling has ceased, other underlying, and less understood causes of decline are still continuing.

Burgeoning anthropogenic threats in the Arabian Sea, including entanglement in fishing gear and ship strikes [6], [94], are known limitations to demographic recovery (e.g.[11]). Future studies should be aimed at understanding in detail which processes have led (or are leading) to the high observed genetic differentiation and more importantly, to the decline of this unique whale population.

## Supporting Information

Figure S1 **Arabian Sea Humpback whales photographed in Dhofar, Southern Oman.** Photo credits: T. Collins and D. MacDonald.
(TIF)

Table S1 **Statistical values obtained in the Bayesian population structure analysis implemented in STRUCTURE using different priors: a) No admixture and prior information on origin of population; b) No admixture and no prior information on origin of population.**
(XLSX)

Table S2 **Estimated contemporary effective population size and number of breeders obtained with the linkage disequilibrium and heterozygote excess methods in the program NeEstimator V2 for Southern Hemisphere humpback whale populations.**
(XLSX)
