# Supplementary figures and images for: The World's Most Isolated and Distinct Whale Population? Humpback Whales of the Arabian Sea
Source: PLoS One. 2014 Dec 3;9(12):e114162. doi: 10.1371/journal.pone.0114162 (PMC4254934; doi:10.1371/journal.pone.0114162)

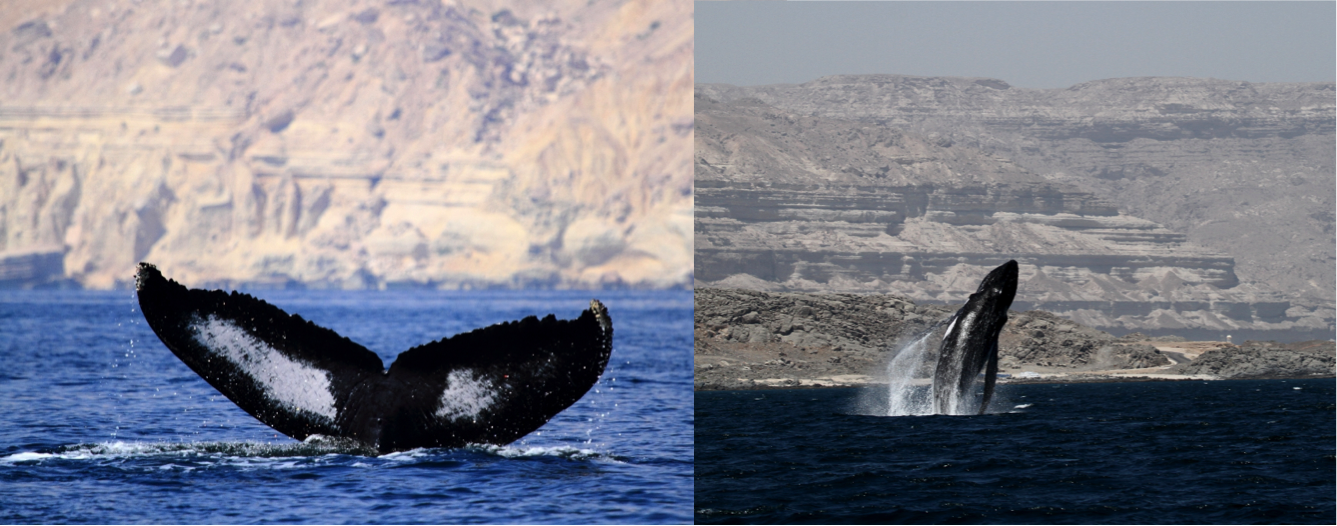

Supplement: Figure S1 — Arabian Sea Humpback whales photographed in Dhofar, Southern Oman. Photo credits: T. Collins and D. MacDonald. (TIF) [file pone.0114162.s001.tif]
